# Metabolites of an Epac-Selective cAMP Analog Induce Cortisol Synthesis by Adrenocortical Cells through a cAMP-Independent Pathway

**DOI:** 10.1371/journal.pone.0006088

**Published:** 2009-06-30

**Authors:** Judith A. Enyeart, John J. Enyeart

**Affiliations:** Department of Neuroscience, The Ohio State University, College of Medicine and Public Health, Columbus, Ohio, United States of America; Flinders University, Australia

## Abstract

Adrenal zona fasciculata (AZF) cells express a cAMP-activated guanine nucleotide exchange protein (Epac2) that may function in ACTH-stimulated cortisol synthesis. Experiments were done to determine whether cAMP analogs that selectively activate Epacs could induce cortisol synthesis and the expression of genes coding for steroidogenic proteins in bovine AZF cells. Treatment of AZF cells with the Epac-selective cAMP analog (ESCA) 8CPT-2′-OMe-cAMP induced large (>100 fold), concentration-dependent, delayed increases in cortisol synthesis and the expression of mRNAs coding for the steroid hydroxylases CYP11a1, CYP17, CYP21, and the steroid acute regulatory protein (StAR). However, a non-hydrolyzable analog of this ESCA, Sp-8CPT-2′-OMe-cAMP, failed to stimulate cortisol production even at concentrations that activated Rap1, a downstream effector of Epac2. Accordingly, putative metabolites of 8CPT-2′-OMe-cAMP, including 8CPT-2′-OMe-5′AMP, 8CPT-2′-OMe-adenosine, and 8CPT-adenine all induced cortisol synthesis and steroid hydroxylase mRNA expression with a temporal pattern, potency, and effectiveness similar to the parent compound. At concentrations that markedly stimulated cortisol production, none of these metabolites significantly activated cAMP-dependent protein kinase (PKA). These results show that one or more metabolites of the ESCA 8CPT-2′-OMe-cAMP induce cortico-steroidogenesis by activating a panel of genes that code for steroidogenic proteins. The remarkable increases in cortisol synthesis observed in this study appear to be mediated by a novel cAMP-, Epac- and PKA-independent signaling pathway.

## Introduction

Cortisol is synthesized from cholesterol and secreted by adrenal zona fasciculata (AZF) cells in a diurnal rhythm under the control of the pituitary peptide adrenocorticotropic hormone (ACTH) [1]. Superimposed on this oscillatory pattern of secretion, stress-induced activation of the hypothalamic-pituitary-adrenal axis leads to additional ACTH-stimulated cortisol production [2]. In AZF cells, the effects of ACTH are mediated through an MC2R melanocortin receptor whose activation is coupled to cAMP synthesis through G_s_ [3]–[5].

ACTH stimulates corticosteroid synthesis through two temporally distinct cAMP-dependent processes. The acute phase occurs within minutes, is independent of gene transcription, and is initiated by the mobilization and delivery of cholesterol to intracellular sites where steroidogenic enzymes are located [1], [6], [7]. The delayed increase in cortisol synthesis is mediated through the enhanced transcription of genes coding for steroidogenic proteins, including the steroidogenic acute regulatory protein (StAR) which transfers cholesterol from the outer to the inner mitochondrial membrane, and the steroidogenic enzymes that mediate the stepwise conversion of cholesterol to cortisol [1], [6], [8]–[10]. Most of these enzymes belong to the cytochrome P450 family of mixed function oxidases [11]. cAMP increases mRNA coding for these proteins within hours, with a temporal pattern resembling that produced by ACTH [1], [6], [8], [9], [12], [13].

Until recently, nearly all of the actions of cAMP in eukaryotic cells were believed to be mediated through the activation of cAMP-dependent protein kinase (PKA). However, two cAMP-activated guanine nucleotide exchange factors (Epac1 and Epac2, also known as cAMP-GEFI and cAMP-GEFII) that activate the small GTPases Rap1 and Rap2 have been identified [14], [15]. Epac1 and Epac2 function in various cellular processes, including the secretion of peptide hormones and neurotransmitters [16]–[18]. While Epac1 is widely expressed mammals, the expression of Epac2 is restricted to a few tissues including the adrenal gland [14], [15].

In this regard, the adrenal gland consists of an inner medulla composed of neural crest-derived chromaffin cells and an outer cortex that includes corticosteroid-secreting cells of mesodermal origin [2]. The adrenal cortex includes aldosterone-secreting zona glomerulosa cells and cortisol-secreting cells of the zona fasciculata (AZF). Although Epac2 is highly expressed in AZF cells [19], it isn't known if cAMP-dependent activation of Epac2 might contribute to ACTH-stimulated cortisol production.

The steroidogenic actions of cAMP, including the rapid and delayed stimulation of cortisol synthesis, have long been attributed to the activation of PKA; however, confounding evidence exists. Specifically, genes that code for steroidogenic enzymes in AZF cells are induced by cAMP, yet nearly all of these, regardless of species, lack consensus cAMP response elements in the 5′ flanking region and therefore do not bind PKA phosphorylated transcription factors [8], [20]–[24].

Further, PKA-stimulated transcription typically occurs with rapid kinetics and does not require *de novo* protein synthesis [25]. In contrast, ACTH- or cAMP-induced increases in steroid hydroxylase-specific mRNAs are cycloheximide sensitive and require periods of up to several hours before they are observed [8], [20], [25]. Therefore, ACTH and cAMP may increase the transcription of steroidogenic enzymes, and stimulate cortisol synthesis by novel PKA-independent mechanisms.

In this regard, PKA-independent actions of ACTH and cAMP have been reported in AZF cells. cAMP inhibits bTREK-1 K^+^ channels in AZF cells by a mechanism that is independent of PKA, but mimicked by Epac-specific cAMP analogs [19], [26]. ACTH enhances the expression of T-type Ca^2+^ channels in rat AZF cells by an H-89 insensitive mechanism [27].

Elucidating the signaling pathways by which ACTH and cAMP regulate corticosteroid synthesis has been hampered by the lack of specific reagents that could independently modulate PKA and Epacs. Exploiting differences in the cAMP-binding domains of these proteins, rational drug design has recently been used to synthesize cAMP derivatives that, at appropriate concentrations, selectively activate Epacs or PKA. These derivatives provide the opportunity to independently study cAMP-activated proteins in the regulation of cell function [28]–[30].

One of these, 8CPT-2′-OMe-cAMP, selectively activates Epacs over PKA, and has been used extensively to determine the role of Epac1 and Epac2 in cell function [28], [30]. However, in the present study we found that, in AZF cells, 8CPT-2′-OMe-cAMP stimulated large, delayed increases in the expression of steroid hydroxylase mRNAs and cortisol synthesis by an Epac-independent mechanism. These effects were mediated by one or more metabolites of the parent compound through an unknown signaling pathway.

## Methods

### Materials

DMEM/F12, antibiotics, fibronectin, and fetal bovine sera (FBS) were obtained from Invitrogen (Carlsbad, CA). Ultrahyb was purchased from Ambion (Austin, TX) and Prime-It II kit for random priming was purchased from Stratagene (La Jolla, CA). Phosphate buffered saline (PBS), tocopherol, selenite, ascorbic acid, and ACTH (1–24) were obtained from Sigma (St. Louis. MO). 8-(4-chlorophenylthio)-2′-O-methyl-cAMP (8CPT-2′-OMe-cAMP -Biolog #C041), hydrolysis-resistant 8-(4-chlorophenylthio)-2′-O-methyl-cAMPs, Sp-isomer (Sp-8CPT-2′-OMe-cAMP - Biolog # C052), 8-(4-chlorophenylthio)-2′-O-methyladenosine-5′-O-monophosphate (8CPT-2-OMe-5′AMP – Biolog # C078), 8-(4-chlorophenylthio)-2′-O-methyladenosine (8CPT-2′OMe-Ado – Biolog #C070), and 8-(4-chlorophenylthio) adenine (8CPT-Ade – Biolog #C069) were purchased from Axxora,LLC (San Diego, CA). Cortisol EIA kit was from Diagnostic Systems Laboratories (Webster, TX). Rap1 activation assay kit was purchased from Millipore (Billerica, MA) and SignaTECT cAMP-dependent protein kinase assay kit was from Promega (Madison, WI).

### Isolation and Culture of AZF Cells

Bovine adrenal glands were obtained from steers (age 2–3 yr) at a local slaughterhouse. AZF cells were isolated as previously described [31]. After isolation, cells were either resuspended in DMEM/F12 (1∶1) with 10% FBS, 100 U/ml penicillin, 0.1 mg/ml streptomycin, and the antioxidants 1 µM tocopherol, 20 nM selenite and 100 µM ascorbic acid (DMEM/F12+) and plated for immediate use, or resuspended in FBS/5% DMSO, divided into aliquots, and stored in liquid nitrogen for future use. To ensure attachment when plating cells, dishes were treated with fibronectin (10 µg/ml) for 30 minutes then rinsed with warm, sterile PBS before adding cells. Cells were maintained at 37°C in a humidified atmosphere of 95% air-5% CO_2_. For experiments where cortisol alone was measured, cells were plated at a density of 0.5–1.0×10^6^ cells/35 mm dish. For experiments where cortisol was measured and RNA was isolated from cell lysates, cells were plated at a density of 5–7×10^6^ cells/60 mm culture dish. For all experiments except those for Rap1, cells were plated 24 h before incubating with ESCAs or metabolites. For Rap1 assay, cells were plated 48 h before incubating with 8CPT-2′-OMe-cAMP or Sp-8CPT-2′-OMe-cAMP.

### Cortisol Assay

Media from experiments was either assayed immediately after collection or frozen (−20°C) until all samples were available. Each experimental condition was assayed in duplicate and duplicate media samples from each were measured using a Cortisol EIA (DSL-10-200) from Diagnostic Systems Laboratories (Webster, TX), according to the manufacturer's directions. If necessary, media samples were diluted using DMEM/F12+. Cortisol values are expressed as mean±SEM of duplicate independent determinations, assayed in duplicate.

### Northern Blot and Measurement of mRNA

Total RNA isolation and Northern blot procedures have been described previously [32]. Briefly, 5–7×10^6^ AZF cells were plated on 60-mm fibronectin-treated dishes in DMEM/F12+. After 24 h, the serum-supplemented media were removed and replaced with either control media (DMEM/F12+) or the same media containing ACTH (1–24), 8CPT-2′-OMe-cAMP, Sp-8CPT-2′-OMe-cAMP, or other agents as required. At the end of the incubation period, total RNA was extracted using RNeasy columns (Qiagen, Valencia, CA), electrophoresed on a denaturing gel, and transferred to a nylon transfer membrane (GeneScreen Plus, PerkinElmer Life Sciences, Waltham, MA). Probes were generated by RT-PCR using AMV reverse transcriptase (Promega, Madison WI), specific primers, and total RNA isolated from bovine AZF cells as described above. Specific probes generated were as follows: StAR- bases 104-1047 of NM_174189, CYP11a1- bases 679-1816 of NM_176644, CYP17 - bases 68-755 of NM_174303, CYP21 – bases 491-1240 of NM_001013596. Probes were labeled with [α-^32^P]dCTP by random primer labeling (Prime-It II, Stratagene, La Jolla, CA). Northern autoradiograms were imaged using a Typhoon 9200 variable mode imager and quantitated using ImageQuant TL v2003.3 software (GE Healthcare Life Sciences, Piscataway, NJ). mRNA values are presented as mean±SEM of at least 3 independent determinations. For the figures, a representative Northern blot is shown for each set of experiments.

### Rap1 Activation Assay

Rap1 activation assays using a glutathione reductase-tagged fusion protein corresponding to amino acids 788–884 of the human Ral-GDS-Rap binding domain bound to glutathione agarose (Ral GDS-RBD agarose) were performed using a Rap1 activation assay kit according to the manufacturer's directions (Millipore, Billerica, MA). Briefly, ∼10×10^6^ cells were plated on fibronectin-treated 10 cm plates in DMEM/F12+ for 48 h, changed to serum-free DMEM for 12 hours, directly stimulated with Epac activators for 15 min, washed twice with ice-cold TBS, then lysed in 600 µl of buffer containing 0.05 M Tris/HCl (pH 7.4), 0.5 M NaCl, 1% NP-40, 2.5 mM MgCl_2_, 5% glycerol, and protease inhibitors (Complete, EDTA-free, Roche Applied Science, Mannheim, Germany). Cells were disrupted by passing lysate five times through a 29-gauge needle; lysate was cleared by centrifugation at 14 000 g for 10 min at 4°C. An aliquot (60 µl) of the supernatant was reserved for estimation of total Rap1. Remaining supernatant was mixed with glutathione-agarose and incubated for 1 h at 4°C. Samples were then centrifuged at 10 000 g for 30 s at 4°C, washed 3× with lysis buffer, suspended in 40 µl of 2× Laemmle buffer containing 10 mM DTT, and separated by 8–16% SDS-PAGE gel electrophoresis. Proteins were transferred to polyvinylidene fluoride (PVDF) membrane, incubated with polyclonal anti-Rap1 antibodies (Millipore, Billerica, MA), and visualized by enhanced chemilluminescence (Pierce of Thermo Fisher Scientific, Rockford, IL).

### A-kinase Assay

PKA activity was measured with a SignaTECT cAMP-dependent protein kinase assay kit (Promega, Madison, WI) in which PKA-dependent phosphorylation of biotinylated peptides can be quantitatively measured as a function of PKA activity. AZF cells were plated on 60-mm fibronectin-treated dishes in DMEM/F12+ at a density of 4×10^6^ cells/dish. After 24 h, the media was replaced with either control media (DMEM/F12+) or the same media containing ACTH (1–24), 8CPT-2′-OMe-cAMP, Sp-8CPT-2′-OMe-cAMP, or other agents as required. At the end of the incubation period, cells were washed 4× with ice-cold PBS, suspended in cold extraction buffer (25 mM Tris-HCl pH 7.4, 0.5 mM EGTA, 10 mM β-mercaptoethanol, 0.5 mM Pefabloc-SC (Roche Applied Science, Indianapolis, IN), and protease inhibitors with EDTA (Complete Mini protease inhibitor cocktail tablet (Roche Applied Science, Indianapolis, IN), 1 per 10 mls lysis solution). Lysates were homogenized using a cold Dounce homogenizer, then centrifuged for 5 min at 4°C, 14,000× *g*. Each experimental condition was assayed in duplicate, and duplicate 5 µl samples of lysate supernatant from each were tested for PKA activity according to the manufacturer's directions. Maximum activatable PKA activity was obtained for each respective sample by the addition of cAMP (5 µM) directly to the sample lysates before determining PKA activity according to the manufacturer's directions. PKA values are presented as mean±SEM.

## Results

### 8CPT-2′-OMe-cAMP induces a delayed increase in Cortisol Secretion by AZF Cells

**Figure 1:**
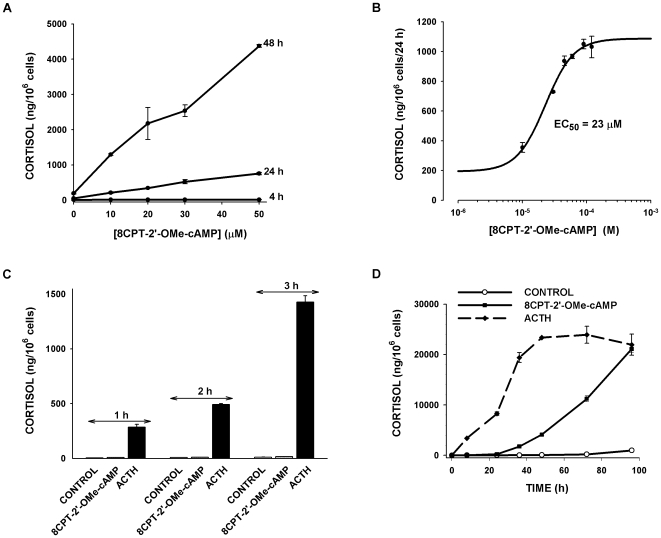
Time- and Concentration-dependent Effects of ACTH and 8CPT-2′-OMe-cAMP on Cortisol Secretion. Bovine AZF cells were plated as described in the Methods. After 24 h, media was replaced with the same media without (control) or with 8CPT-2′-OMe-cAMP or ACTH. A) Time- and concentration-dependent effects of 8CPT-2′-OMe-cAMP on cortisol secretion. Media was sampled and cortisol measured at 4, 24 and 48 h after treating AZF cells with 8CPT-2′-OMe-cAMP at concentrations ranging from 10 to 50 µM. B) Concentration-response curve for 8CPT-2′-OMe-cAMP-stimulated on cortisol secretion after 24 h. Data were fit with an equation of the form: Y = min+(max−min)/(1+10^∧^((Log EC_50_−X)*b)) where EC_50_ is the concentration that produces 50% of maximal effect and *b* is the Hill slope. C) Effect of 8CPT-2′-OMe-cAMP (30 µM) and ACTH (2 nM) on cortisol secretion at 1, 2 or 3 h. D) Time course for ACTH (2 nM) and 8CPT-2′-OMe-cAMP (30 µM). Media was sampled and cortisol determined at times from 8 to 96 h.

When applied to bovine AZF cells, the ESCA 8CPT-2′-OMe-cAMP produced a remarkable concentration-dependent, delayed increase in cortisol secretion. As illustrated in Figure 1A, a 4 h exposure to 8CPT-2′-OMe-cAMP at concentrations between 10 and 50 µM produced little or no increase in the quantity of cortisol secreted. However, by 24 h, large 4 to 15 fold increases in the quantity of cortisol produced were present. Between 24 and 48 h, the ESCA induced even greater increases in the rate of secretion; by 48 h, 8CPT-2′-OMe-cAMP (50 µM) had increased the quantity of cortisol produced more than 20 times the control value. 8CPT-2′-OMe-cAMP stimulated cortisol synthesis with an EC_50_ of 23 µM (Figure 1B).

ACTH stimulates cortisol secretion through rapid and delayed cAMP-dependent mechanisms that are incompletely understood [1], [13]. Experiments with 8CPT-2′-OMe-cAMP suggested that the delayed effects of ACTH on cortisol synthesis could be mediated through activation of Epac2. ACTH was compared to 8CPT-2′-OMe-cAMP with respect to the time-dependent stimulation of cortisol production for periods from 1 to 96 h. While ACTH-induced increases in the rate of cortisol secretion were present by 1 h and subsided by 48 h, the delayed 8CPT-2′-OMe-cAMP-stimulated increases in the rate of cortisol synthesis persisted for at least 96 h (Figure 1C,D).

### 8CPT-2′-OMe-cAMP Stimulates Expression of Steroid Hydroxylase mRNAs

**Figure 2:**
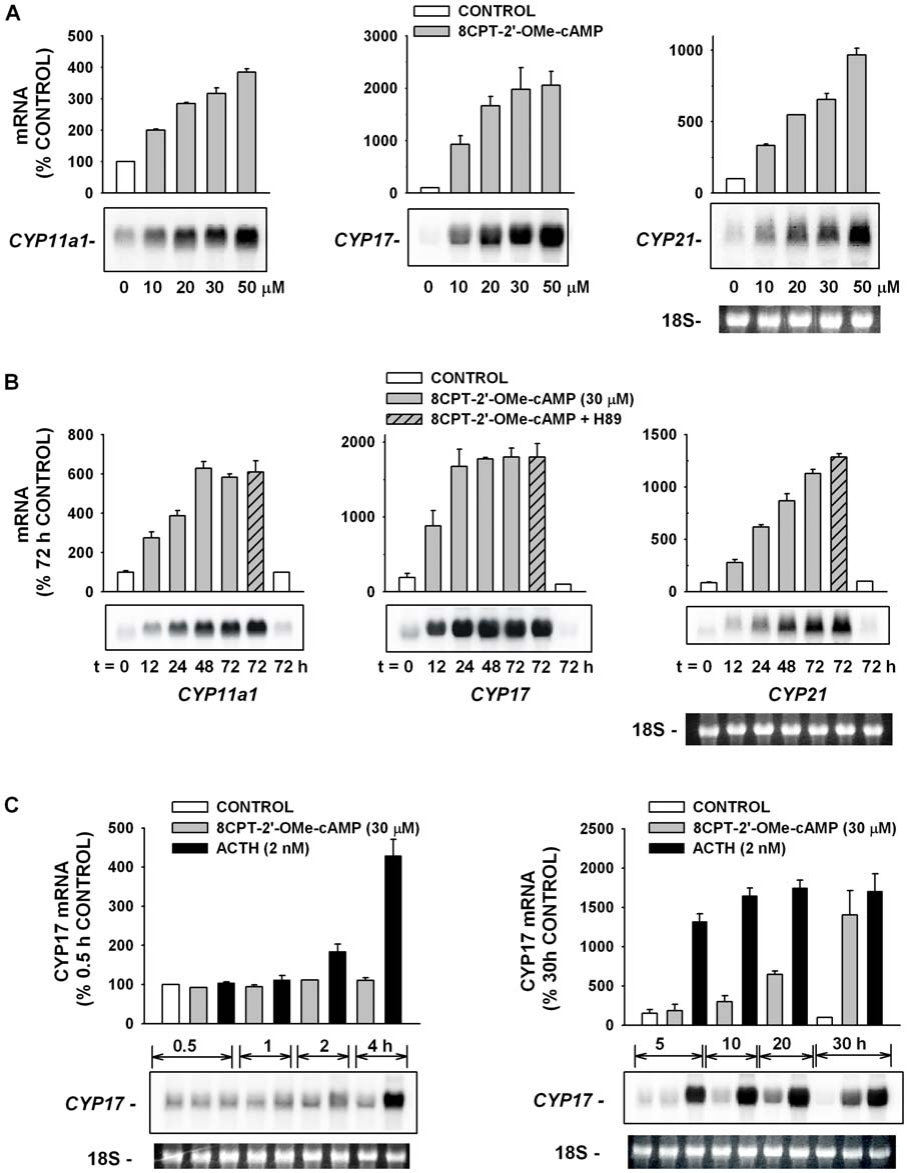
Effects of ACTH and 8CPT-2′-OMe-cAMP on Expression of Genes Coding for Steroidogenic Proteins. AZF cells were incubated either without (control), or with 8CPT-2′-OMe-cAMP, 8CPT-2′-OMe-cAMP plus H-89, or ACTH as indicated. Total RNA was isolated at indicated times. Each lane contained 10 µg of total RNA. Membranes were hybridized with specific probe for bovine CYP11a1, then stripped and probed for CYP17, stripped and reprobed for CYP21, as indicated. 18S rRNA bands from representative gels are shown as evidence of even loading. A) Concentration-dependent effect of 8CPT-2′-OMe-cAMP on CYP11a1, CYP17, and CYP21 mRNA. AZF cells were either untreated (control, white bar) or treated with 10–50 µM 8CPT-2′-OMe-cAMP (grey bars) for 48 hr before isolating total RNA. B) Time-dependent effect of 8CPT-2′-OMe-cAMP on CYP11a1, CYP17, and CYP21. AZF cells were incubated without (control, white bar), or with 8CPT-2′-OMe-cAMP (30 µM) (grey bars), or pre-incubated with H-89 (10 µM) for 30 min before adding 8CPT-2′-OMe-cAMP (30 µM) (striped/grey bar) for times indicated, after which total RNA was isolated. C) Comparison of time-dependent effects of 8CPT-2′-OMe-cAMP (30 µM) and ACTH (2 nM) on CYP17 mRNA. AZF cells were incubated either without (control, white bar) or with 8CPT-2′-OMe-cAMP (30 µM, grey bar) or ACTH (2 nM, black bar) for 0.5 to 4 h (left panel) or 5 to 30 h (right panel), after which total RNA was isolated.

The delay of many hours that preceded 8CPT-2′-OMe-cAMP-stimulated cortisol secretion suggested that the response was mediated through an action on gene transcription. CYP11a1, CYP17, and CYP21 are three of the steroid hydroxylases that mediate the stepwise synthesis of cortisol from cholesterol. CYP11a1 is a mitochondrial enzyme that catalyses the synthesis of pregnenolone from cholesterol, the first and rate-limiting enzymatic step in cortisol synthesis. CYP17 and CYP21 are microsomal enzymes [6]. 8CPT-2′-OMe-cAMP (10-50 µM) induced concentration-dependent increases in the expression of each of the three steroid hydroxylase mRNAs. In the experiment illustrated in Figure 2A, maximum increases ranged from over three fold (3.3±1.1, n = 7) for CYP11a1 to 20 fold (20.6±2.6, n = 6) for CYP17. The increases in CYP17 mRNA were typically greatest relative to time-matched controls since this is the only one of the steroid hydroxylases whose expression is reported to be entirely cAMP-dependent [33], [34]. The 8CPT-2′-OMe-cAMP-stimulated increases in the quantity of mRNAs for each of the steroid hydroxylases occurred only after a delay of more than 4 h, while mRNA levels continued to increase for periods up to 72 h (Figure 2B,C).

The induction of the steroid hydroxylase mRNAs by the ESCA was not reduced by the PKA antagonist H-89. Overall, H-89 (10 µM) did not significantly affect 8CPT-2′-OMe-cAMP-induced CYP17 and CYP11a1 expression (Figure 2B).

The kinetics of ACTH and 8CPT-2′-OMe-cAMP stimulation of steroid hydroxylase mRNA expression were consistent with their actions on cortisol secretion. In the experiments illustrated in Figure 2C, the kinetics of ACTH and 8CPT-2′-OMe-cAMP induction of CYP17 mRNAs were compared at times from 0.5 to 30 h. ACTH induced large increases in CYP17 mRNA that were present after 4 h, and reached a maximum of 20 fold by 20 h. In contrast, 8CPT-2′-OMe-cAMP stimulated a slower, delayed increase in CYP17 mRNA that was significant at 10 h, but had not reached a maximum by 30 h. Similar results were obtained for CYP11a, and CYP21 (data not shown).

### Sp-8CPT-2′-OMe-cAMP does not Stimulate Cortisol Synthesis

**Figure 3:**
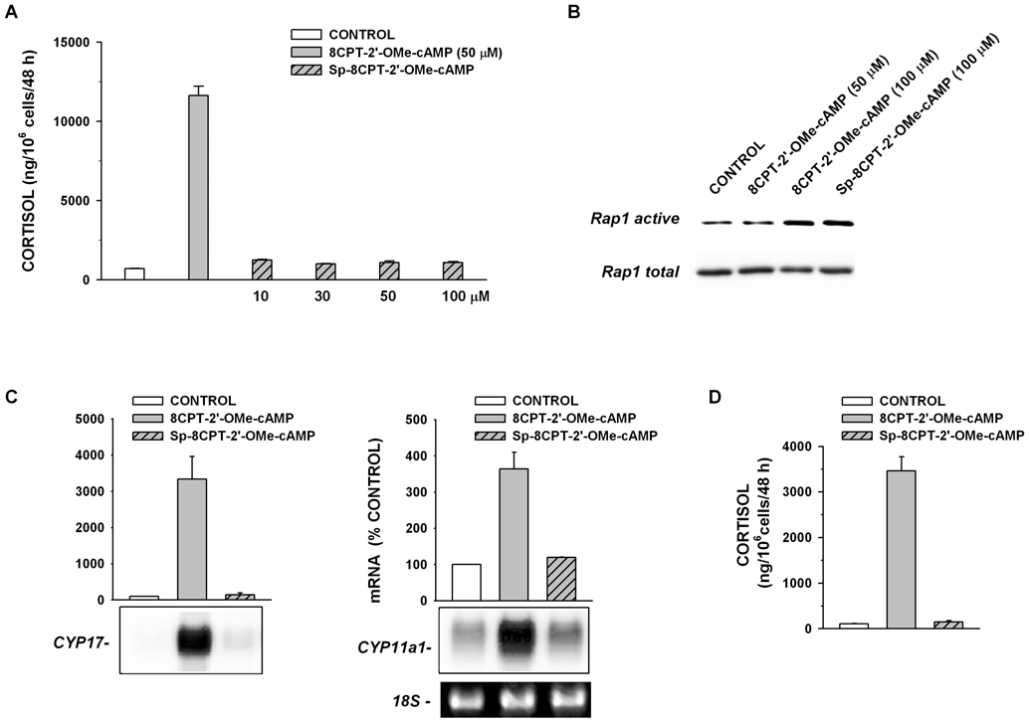
Sp-8CPT-2′-OMe-cAMP activates RAP1A but does not increase either Cortisol Secretion or Steroidogenic Protein Gene Expression. AZF cells were either untreated (control), or incubated with 8CPT-2′-OMe-cAMP, or Sp-8CPT-2′-OMe-cAMP, as indicated. A) Comparison of effect of 8CPT-2′-OMe-cAMP (50 µM) and Sp-8CPT-2′-OMe-cAMP (30, 50, or 100 µM) on cortisol secretion after 48 h. B) Activation of Rap1 by 8CPT-2′-OMe-cAMP and Sp-8CPT-2′-OMe-cAMP in AZF cells. For pull-down assays, AZF cells were prepared and cultured in 10 cm dishes as described in the Methods. Before lysis, cells were incubated for 15 min either without (control) or with 50 or 100 µM 8CPT-2′-OMe-cAMP, or 100 µM SP-8CPT-2′-OMe-cAMP. C) Sp-8CPT-2′-OMe-cAMP does not increase CYP17 or CYP11a1 mRNA expression. AZF cells were incubated without (control, white bar) or with either 8CPT-2′-OMe-cAMP (30 µM, grey bar), or Sp-8CPT-2′-OMe-cAMP (30 µM, striped/grey bar). Total RNA was isolated after 48 h, electrophoresed, blotted and probed as described in the Methods. Each lane contained 10 µg of total RNA. Membranes were hybridized with specific probe for bovine CYP11a1 or CYP17, as indicated. D) Cortisol measurements from media samples from experiment illustrated in (C).

The ability of 8CPT-2′-OMe-cAMP to induce large increases in the expression of steroid hydroxylase mRNAs and cortisol synthesis by bovine AZF cells suggested that the delayed actions of ACTH and cAMP on cortico-steroidogenesis are mediated through activation of Epac2. However, similar to cAMP, this ESCA is metabolized by cyclic nucleotide phosphodiesterase as well as other enzymes [30]. To determine whether the steroidogenic actions of 8CPT-2′-OMe-cAMP were mediated by this compound rather than a metabolite, we measured the effect of the poorly hydrolyzable analog Sp-8CPT-2′-OMe-cAMP on cortisol synthesis and the expression of steroid hydroxylase mRNAs. Although Sp-8CPT-2′-OMe-cAMP activates Epac proteins with a potency similar to its hydrolyzable analog [30], [35], the non-hydrolyzable compound at concentrations up to 100 µM was ineffective at increasing cortisol secretion or enhancing the expression of steroid hydroxylase mRNAs (Figure 3A,C,D). By comparison, at 100 µM, 8CPT-2′-OMe-cAMP and its non-hydrolyzable analog activated Rap1 similarly (Figure 3B).

These results indicated that the substantial increases in steroid hydroxylase mRNAs and cortisol secretion induced by 8CPT-2′-OMe-cAMP were not mediated through Epac2. They further suggested that this response required the conversion of this ESCA to one or more active metabolites.

### Effect of Metabolites of 8CPT-2′-OMe-cAMP on Steroidogenesis

**Figure 4:**
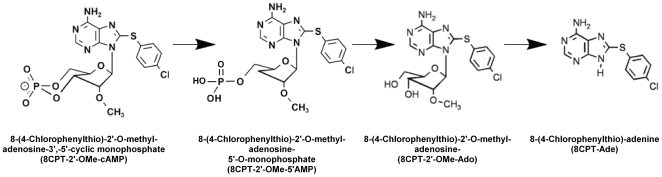
Structures of 8CPT-2′-OMe-cAMP and Metabolites. Chemical structures of 8CPT-2′-OMe-cAMP and its metabolites.

**Figure 5:**
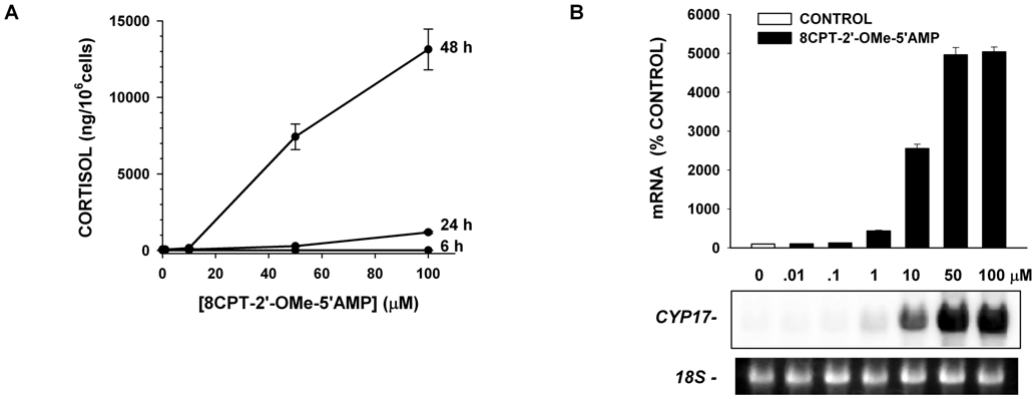
The 8CPT-2′-OMe-cAMP Metabolite 8CPT-2′-OMe-5′AMP Increases Cortisol Secretion and CYP17 mRNA Expression. A) Time- and concentration-dependent effects of 8CPT-2′-OMe-5′AMP on cortisol secretion by AZF cells. AZF cells were treated with 8CPT-2′-OMe-5′AMP at concentrations ranging from 0.01 to 100 µM. Cortisol was measured from media samples after 6, 24 and 48 h. B) Concentration-dependent effects of 8CPT-2′-OMe-5′AMP on CYP17 mRNA expression. AZF cells were incubated without (control, white bar) or with 0.01–100 µM 8CPT-2′-OMe-5′AMP (black bars) for 48 hr before isolating total RNA.

8CPT-2′-OMe-cAMP can be converted to 8CPT-2′-OMe-5′AMP by cyclic nucleotide phosphodiesterase (see Figure 4) [30], [36]. This metabolite stimulated cortisol secretion and expression of steroid hydroxylase with a temporal pattern, potency, and effectiveness similar to the parent ESCA (Figure 5). In the experiment illustrated, a 6 h exposure to 8CPT-2′-OMe-5′AMP (100 µM) did not significantly increase cortisol synthesis. However, by 48 h, cortisol production increased 400 fold. Accordingly, after 48 h, 8CPT-2′-OMe-5′AMP (50 µM) had induced a nearly 50 fold (49.6±1.9, n = 3) increase in CYP17. CYP11a1 and CYP21 mRNAs were also increased similarly (data not shown).

**Figure 6:**
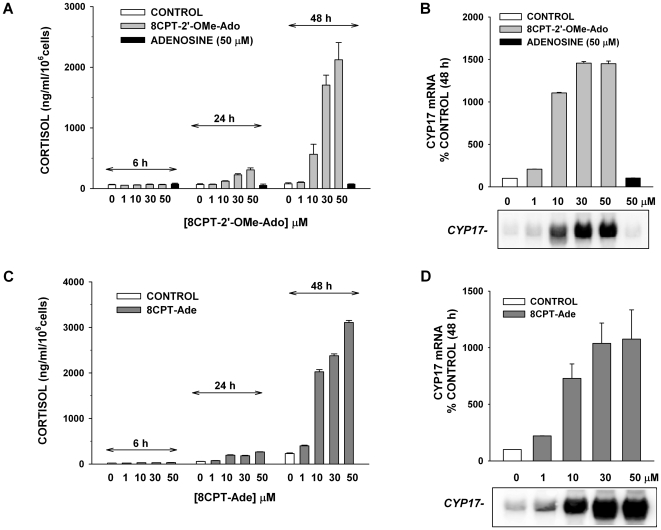
8CPT-2′-OMe-Adenosine and 8CPT-Adenine Increase Cortisol Secretion and CYP17 mRNA Expression. AZF cells were incubated either without (control), or with 8CPT-2′-OMe-Ado, adenosine, or 8CPT-Ade at concentrations from 1-50 µM. Media was sampled for cortisol measurements at several time points and total RNA was isolated after 48 h for measurement of CYP17 mRNA. A,B) Time- and concentration-dependent effects of 8CPT-2′-OMe-Ado on cortisol secretion and CYP17 mRNA. A) Media was sampled and cortisol measured at 6, 24 and 48 h after incubating AZF cells without (control, white bars) or with 8CPT-2′-OMe-Ado at concentrations ranging from 1 to 50 µM (grey bars) or adenosine (50 µM, black bar). B) After 48 h, mRNA was isolated and CYP17 mRNA measured by Northern blot. C, D) Time- and concentration-dependent effects of 8CPT-Ade on cortisol secretion and CYP17 mRNA. C) Media was sampled and cortisol determined at 6, 24 and 48 h after treating AZF cells without (control, white bars) or with 8CPT-Ade at concentrations ranging from 1 to 50 µM (dark grey bars). D) After 48 h, mRNA was isolated and CYP17 mRNA was measured by Northern blot.

8CPT-2′-OMe-5′AMP itself may be a substrate for 5′ nucleotidases that would convert this molecule to 8-(4-chlorophenylthio)-2′-O-methyladenosine (8CPT-2′-OMe-Ado) (see Figure 4) [36]. Accordingly, 8CPT-2′-OMe-Ado stimulated cortisol secretion and the expression of steroid hydroxylases with characteristics similar to the parent molecules. In contrast, adenosine was completely ineffective at stimulating cortisol synthesis or mRNA expression (Figure 6A,B).

8CPT-2′-OMe-Ado may be further metabolized to 8CPT-2′-adenine (8CPT-Ade) by purine nucleoside phosphorylase (see Figure 4) [36]. 8CPT-Ade also induced a delayed increase in both cortisol synthesis and expression of steroid hydroxylase mRNA with a temporal pattern, potency, and effectiveness similar to each of the previously described putative metabolites (Figure 6C, D).

In summary, the Epac-selective cAMP activator 8CPT-2′-OMe-cAMP stimulated cortico-steroidogenesis by bovine AZF cells through a mechanism which is likely independent of Epac2 activation. Rather, the steroidogenic effect appears to be mediated through one or more metabolites that, after several hours delay, induce the expression of mRNAs coding for steroid hydroxylases involved in the synthesis of cortisol from cholesterol.

### Stimulation of StAR Expression by 8CPT-2′-OMe-5′AMP and its Metabolites

**Figure 7:**
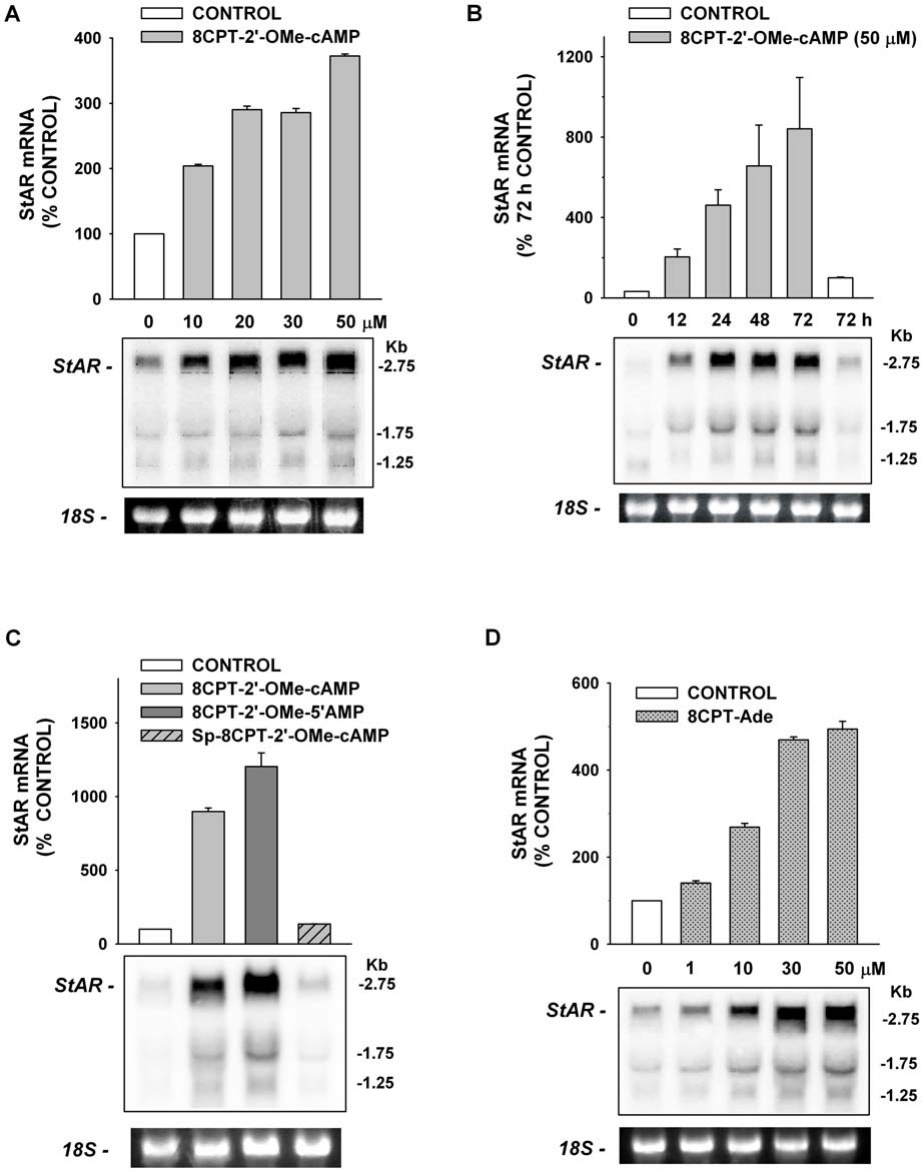
Effects of 8CPT-2′-OMe-cAMP, Metabolites, and Sp-8CPT-2′-OMe-cAMP on StAR mRNA Expression. AZF cells were incubated either without (control), or with 8CPT-2′-OMe-cAMP, 8CPT-2′-OMe-5′AMP, Sp-8CPT-2′-OMe-cAMP, or 8CPT-Ade at indicated times. Total RNA was isolated after indicated times. Membranes were hybridized with specific probe for bovine StAR mRNA as described in the Methods. Each lane contained 10 µg of total RNA. 18S rRNA bands from representative gels are shown as evidence of even loading. A) Concentration-dependent effect of 8CPT-2′-OMe-cAMP on StAR mRNA. AZF cells were incubated without (control, white bar) or with 10–50 µM 8CPT-2′-OMe-cAMP (grey bars) for 48 hr before isolating total RNA. B) Time-dependent effects of 8CPT-2′-OMe-cAMP on StAR mRNA. AZF cells were incubated without (control, white bar), or with 8CPT-2′-OMe-cAMP (50 µM, grey bars) for indicated times after which total RNA was isolated. mRNA is expressed as % control value at 72 h. C) Comparison of effects of 8CPT-2′-OMe-cAMP, 8CPT-2′-OMe-5′AMP, and hydrolysis-resistant Sp-8CPT-2′-OMe-cAMP on StAR mRNA. AZF cells were incubated without (control, white bar) or with either 8CPT-2′-OMe-cAMP (30 µM, light grey bar), 8CPT-2′-OMe-5′AMP (30 µM, dark grey bar), or Sp-8CPT-2′-OMe-cAMP (30 µM, striped/grey bar) for 48 h after which total RNA was isolated. D) Concentration-dependent effect of 8CPT-Ade on StAR mRNA. AZF cells were incubated without (control, white bar) or with 10–50 µM 8CPT-Ade (grey bars) for 48 hr before isolating total RNA.

The rate-limiting step in ACTH-stimulated cortisol synthesis, the transfer of cholesterol from the outer to the inner mitochondrial membrane, is mediated by StAR [7], [10], [37]. We found that, in addition to increasing the expression of steroid hydroxylase genes, 8CPT-2′-OMe-cAMP also induced the expression of StAR mRNA. The potency and temporal pattern were similar to those observed for increases in steroid hydroxylase mRNAs (Figure 7A,B). In contrast, Sp-8CPT-2′-OMe-cAMP failed to induce the expression of StAR mRNA, indicating that this effect was again mediated by one or more metabolites of the hydrolyzable ESCA (Figure 7C). Accordingly, both 8CPT-2′-OMe-5′AMP and 8CPT-adenine stimulated large increases in the quantity of StAR mRNAs (Figure 7C,D). 8CPT-2′-OMe-cAMP and 8CPT-Ade induced StAR mRNA similarly at concentrations from 10–50 µM (Figure 7A,D).

### Steroidogenic Effects of 8CPT-2′-OMe-cAMP are PKA-independent

Although 8CPT-2′-OMe-cAMP is a relatively specific activator of Epac proteins, it is a weak and less effective activator of PKA [28], [30], [35]. It isn't known whether the putative Epac metabolites activate PKA at the same concentrations that stimulate cortisol secretion.

**Figure 8:**
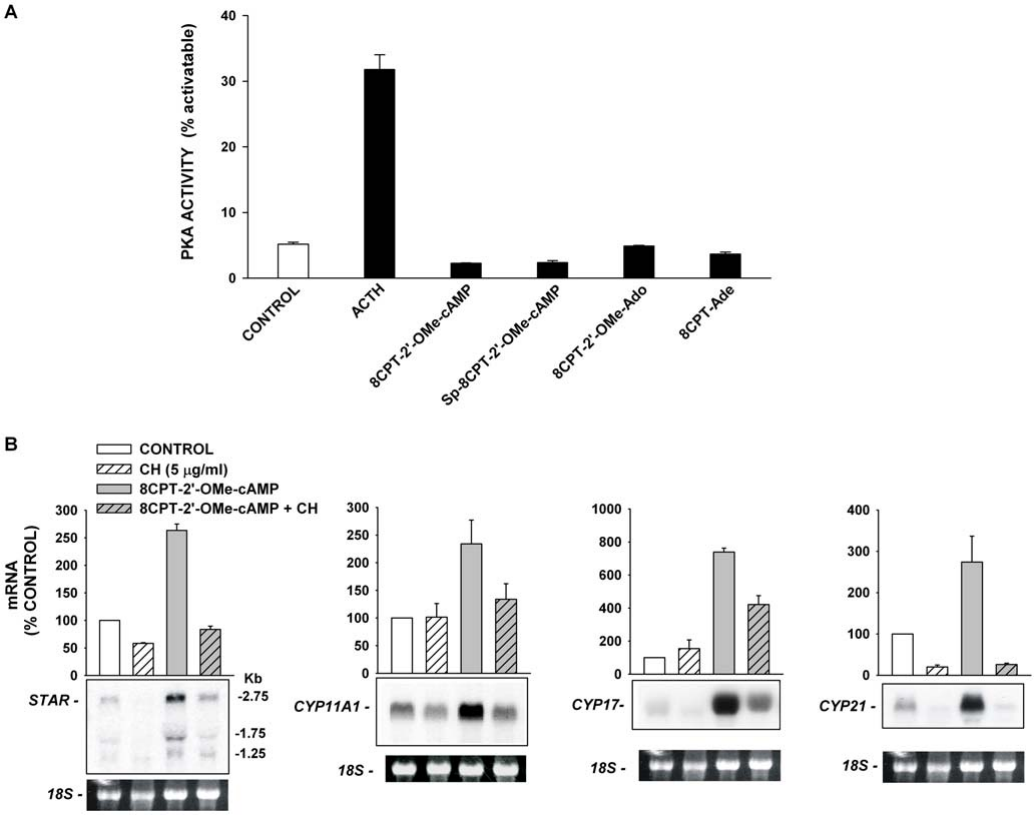
Effects of 8CPT-2′-OMe-cAMP and Metabolites are PKA-independent but Cycloheximide-sensitive. A) Effect on PKA activity. AZF cells were either untreated (control), or incubated with ACTH (2 nM), 8CPT-2′-OMe-cAMP (30 µM), Sp-8CPT-2′-OMe-cAMP (30 µM), 8CPT-2′-OMe-ado (30 µM), or 8CPT-ade (30 µM) for 30 min after which lysates were collected and assayed for PKA activity as described in the Methods. PKA activity is expressed as mean±SEM of duplicate determinations from duplicate independent samples. B) Effect of cycloheximide on 8CPT-2′-OMe-cAMP-dependent increases in steroidogenic and StAR mRNAs. AZF cells were either untreated (control, white bar), or incubated with cycloheximide (CH, 5 µg/ml, striped bar), 8CPT-2′-OMe-cAMP (30 µM, grey bar), or 8CPT-2′-OMe-cAMP plus CH (striped/grey bar), as indicated. Total RNA was isolated after 48 h. Membranes were hybridized with specific probes for StAR, CYP11a1, CYP17, or CYP21 mRNA. Each lane contained 10 µg of total RNA. StAR mRNA value was determined from % control for the 2.75 Kb band value.

It was discovered that none of these agents increased PKA activity in AZF cells when they were applied at concentrations sufficient to induce large increases in cortisol synthesis (Figure 8A). These metabolites also failed to activate PKA when applied directly to AZF cell lysates (data not shown).

### Cycloheximide Sensitivity

The delay of hours that precedes the 8CPT-2′-OMe-cAMP-induced increase in the expression of genes coding for steroidogenic proteins suggests that this effect requires *de novo* protein synthesis. Accordingly, the protein synthesis inhibitor cycloheximide (5 µg/ml) inhibited the expression of mRNAs coding for each of the three steroid hydroxylases and StAR (Figure 8B).

## Discussion

The major findings of this study are that the Epac-selective cAMP analog 8CPT-2′-OMe-cAMP induced large, delayed increases in the expression of mRNAs coding for steroidogenic proteins, including three steroid hydroxylases and StAR, leading to corresponding increases in cortisol synthesis. Importantly, these corticosteroidogenic actions are not mediated by the ESCA itself, but by one or more enzymatic products of this compound. These metabolites, including 8CPT-2′-OMe-5′AMP, 8CPT-2′-OMe-Ado, and 8CTP-Ade, all induce mRNAs and cortisol synthesis with a potency and temporal pattern similar to the parent compound, but by an unknown Epac2- and PKA-independent signaling pathway. The function of Epac2 in bovine AZF cell physiology remains unknown.

### 8CPT-2′-OMe-cAMP and Metabolites

Initial experiments in which the ESCA induced pronounced, delayed increases in the expression of steroid hydroxylase and StAR mRNAs and associated cortisol synthesis led us to believe that these effects were mediated through activation of Epac2 which is robustly expressed in bovine AZF cells [19]. The failure of Sp-8CPT-2′-OMe-cAMP to mimic the actions of its hydrolyzable analog was unexpected and appears to eliminate the possibility that ACTH- or cAMP-stimulated increases in cortisol synthesis are mediated in part by Epac2.

These results also suggested that 8CPT-2′-OMe-cAMP functions in this instance only after conversion to one or more bioactive metabolites. Accordingly, we found that three potential metabolites of the ESCA mimicked its effects on cortisol secretion and gene expression. Enzymes that could catalyze the conversion of 8CPT-2′-OMe-cAMP to each of these presumptive metabolites are expressed by mammalian cells. These include cyclic nucleotide phosphodiesterases, 5′nucleotidases, hypoxanthine phosphoribosyltransferase, and nucleotide phosphorylases [36].

### Receptors and Signaling Pathways

Currently, the active metabolites, associated receptor, and signaling pathway(s) that mediate the increases in cortisol synthesis and gene expression observed in this study have not been identified. It will be important to learn if 8CPT-Ade is further metabolized to an active compound.

Whatever the identity of the active metabolite, it appears certain that it acts through a signaling pathway that is different from those activated by cAMP. First, in contrast to cAMP which triggers both rapid and delayed increases in cortisol synthesis through PKA-dependent mechanisms, 8CPT-2′-OMe-cAMP and its metabolites stimulate only delayed increases in cortisol without activating PKA [1], [13], [38].

The activation of Rap1 by 8CPT-2′-OMe-cAMP and it non-hydrolyzable analog were not correlated with the expression of steroidogenic mRNAs or cortisol production. In fact, 8CPT-2′-OMe-cAMP stimulated cortisol synthesis at concentrations that produced little or no Rap1 activation, while at higher concentrations, Sp-8CPT-2′-OMe-cAMP activated Rap1 without increasing cortisol synthesis. These results argue against any role for Epac2 in cAMP-stimulated cortico-steroidogenesis.

Overall, the findings of this study do not rule out the possibility that an ESCA metabolite functions through an unknown cAMP-independent pathway which may also be activated by ACTH. ACTH and an *O*-nitrophenyl sulphenyl derivative produce large increases in cortisol synthesis at concentrations that stimulate no measurable increase in cAMP production [39], [40]. Alternatively, metabolite-sensing riboswitches, including those binding adenine and adenine derivatives, control gene expression in prokaryotes as well as eukaryotes [41], [42]. It is not known whether hormone synthesis in eukaryotic cells could also be regulated by small molecules acting through riboswitches. ESCA metabolites could also activate unidentified orphan receptors.

The delay of several hours and the cycloheximide-sensitivity indicate that 8CPT-2′-OMe-cAMP-stimulated mRNA expression and cortisol secretion require *de novo* protein synthesis. It is possible that the active molecule induces the expression of a transcription factor that specifically enhances the transcription of genes coding for steroidogenic proteins. In this regard, we found that 8CPT-2′-OMe-cAMP and its metabolites do not stimulate the expression of mRNAs coding for other AZF cell proteins, including the transcriptional repressor DAX-1 and the Kv1.4 K^+^ channel (unpublished observations). However, the range of genes whose expression might be increased or decreased through this novel signaling pathway is yet to be determined. It will be interesting to compare ACTH and cAMP to 8CPT-Ade with respect to their effects on the expression of the entire transcriptome of bovine AZF cells.

Neither 8CPT-2′-OMe-cAMP nor its presumptive metabolites, at concentrations that triggered large increases in cortisol synthesis, increased overall RNA synthesis or AZF cell proliferation. Rather, these agents had a slight inhibitory effect on proliferation. Therefore, none of the delayed increases in cortisol synthesis could be attributed to increases in the number of AZF cells (unpublished observations).

Since its introduction in 2002, 8CPT-2′-OMe-cAMP has been used in hundreds of studies to determine whether Epacs regulate the function of cellular proteins, including ion channels and enzymes and processes ranging from secretion and cell adhesion to hormone gene expression [16], [28], [30]. The discovery that one or more metabolites of the ESCA 8CPT-2′-OMe-cAMP markedly induce cortisol secretion and the expression of four associated genes indicates that the results of studies in which this compound has been used as a selective activator of Epac proteins may require re-evaluation. Further, to confirm that a cellular response is mediated through Epac1 or Epac2, it should be produced by hydrolyzable as well as non-hydrolyzable Epac activators.

The extent to which metabolites of ESCAs might regulate gene expression and cell function in other tissues and organisms is unknown. Perhaps these agents will regulate the synthesis of other steroid hormones, including the adrenal mineralocorticoid aldosterone, and the gonadal steroids testosterone and estrogen [20]. It has been reported that 8CPT-2′-OMe-cAMP metabolites also inhibit proliferation of the protozoa *Trypanosoma brucei* [43]. It is possible that these responses might involve activation of a common ancient signaling pathway present in both prokaryotes and eukaryotes.
